# Small Airway Dysfunction in Cough Variant Asthma: Prevalence, Clinical, and Pathophysiological Features

**DOI:** 10.3389/fphys.2021.761622

**Published:** 2022-01-13

**Authors:** Fang Yi, Ziyu Jiang, Hu Li, Chunxing Guo, Hankun Lu, Wei Luo, Qiaoli Chen, Kefang Lai

**Affiliations:** State Key Laboratory of Respiratory Disease, National Clinical Research Center for Respiratory Disease, Guangzhou Institute of Respiratory Health, The First Affiliated Hospital of Guangzhou Medical University, Guangzhou, China

**Keywords:** cough variant asthma, small airway dysfunction, prevalence, cough severity, airway inflammation

## Abstract

**Introduction:** Small airway dysfunction (SAD) commonly presents in patients with classic asthma, which is associated with airway inflammation, disease severity, and asthma control. However, the prevalence of SAD, its relationship with cough severity and airway inflammation, and its development after antiasthmatic treatment in patients with cough variant asthma (CVA) need to be clarified. This study aimed to investigate the prevalence of SAD and its relationship with clinical and pathophysiological characteristics in patients with CVA and the change in small airway function after antiasthmatic treatment.

**Methods:** We retrospectively analyzed 120 corticosteroid-naïve patients with CVA who had finished a standard questionnaire and relevant tests in a specialist cough clinic, such as cough visual analog scale (VAS), differential cells in induced sputum, fractional exhaled nitric oxide (FeNO) measurement, spirometry, and airway hyper-responsiveness. Information of 1-year follow-up was recorded in a part of patients who received complete cough relief after 2 months of treatment. SAD was defined as any two parameters of maximal mid-expiratory flow (MMEF)% pred, forced expiratory flow at 50% of forced vital capacity (FEF50%) pred, and forced expiratory flow at 75% of forced vital capacity (FEF75%) pred measuring <65%.

**Results:** SAD occurred in 73 (60.8%) patients with CVA before treatment. The patients with SAD showed a significantly longer cough duration (24.0 vs. 6.0, *p* = 0.031), a higher proportion of women (78.1 vs. 59.6%, *p* = 0.029), older mean age (41.9 vs. 35.4, *p* = 0.005), and significantly lower forced expiratory volume in 1 s (FEV_1_%) pred, FEV_1_/FVC, MMEF% pred, FEF50% pred, FEF75% pred, PEF% pred, and PD20 (all *p* < 0.01) as compared with patients without SAD. There were no significant differences in cough VAS, sputum eosinophils count, FeNO, and TIgE level between patients with SAD and those without SAD. Among 105 patients who completed 2 months of antiasthmatic treatment and repeatedly experienced spirometry measurement, 57 (54.3%) patients still had SAD, despite a significant improvement in cough VAS, sputum eosinophils, FeNO, FEF50% pred, and PEF% pred (all *p* < 0.01). As compared with patients without SAD, patients with SAD showed no significant differences in the relapse rate (50.0 vs. 41.9%, *p* = 0.483) and wheeze development rate (10.4 vs. 0%, *p* = 0.063) during the follow-up.

**Conclusions:** Small airway dysfunction occurred in over half of patients with CVA and persisted after short-term antiasthmatic treatment, which showed distinctive clinical and pathophysiological features.

## Introduction

Cough variant asthma (CVA) is the most common cause of chronic cough, characterized by a cough as a sole or predominant symptom and airway hyper-responsiveness, without obvious wheezing or shortness of breath as seen in classic asthma (Lai et al., 2013, 2018). Previous studies have shown that up to 40% of patients with CVA could develop into classic asthma (Matsumoto et al., 2006). Higher airway responsiveness, higher sputum eosinophils, and atopy are risk factors for the development of CVA into classic asthma (Kim et al., 2003; Fujimura et al., 2004; Takemura et al., 2007). Classic asthma is often accompanied by small airway dysfunction (SAD), at a proportion commonly ranging from 40 to 70% (Tunon-De-Lara et al., 2007; Anderson et al., 2012; Usmani et al., 2016), and is particularly prevalent in severe asthma and difficult-to-treatment asthma (Contoli et al., 2012). Classic asthma patients with SAD have higher fractional exhaled nitric oxide (FeNO) levels, higher eosinophil counts, and higher airway responsiveness, predicting the use of higher doses of inhaled corticosteroids (ICS), poorer response to corticosteroids, and worse asthma control (Manoharan et al., 2014; Van Der Wiel et al., 2014; Kuo et al., 2018; Cottini et al., 2020).

Growing evidence supports that SAD commonly presents in milder asthma (Anderson et al., 2012). For patients with CVA, it was reported that small airway function was lower than that of non-CVA patients with chronic cough, and the increase in FeNO levels was related to the decrease in small airway function (Feng-Jia et al., 2018). Although that study reported the proportion of CVA patients with SAD, its relationship with the severity of symptoms and airway inflammation, and its change after antiasthmatic treatment have not been well-investigated. Thus, we aimed to investigate the prevalence of SAD and its related clinical and pathophysiological characteristics in patients with CVA and the change in small airway function after antiasthmatic treatment.

## Materials and Methods

### Subjects

A total of 120 steroid-naïve patients with CVA who visited the specialist cough clinic of the First Affiliated Hospital of Guangzhou Medical University were enrolled in this study. The diagnostic criteria of CVA were as follows: (1) chronic cough as the sole presenting complaint lasting more than 8 weeks, (2) no abnormalities on chest X-ray; (3) a positive result of methacholine bronchoprovocation test (a positive result of bronchodilator test in two patients); (4) no antiasthmatic treatment, such as ICS, combination of ICS and a long-acting β_2_-agonist (ICS/LABA), or/and leukotriene receptor antagonists (LTRAs) in the past 8 weeks. Patients with upper airway infection in the past 4 weeks or with other serious comorbidities were excluded.

### Study Design

**Figure 1:**
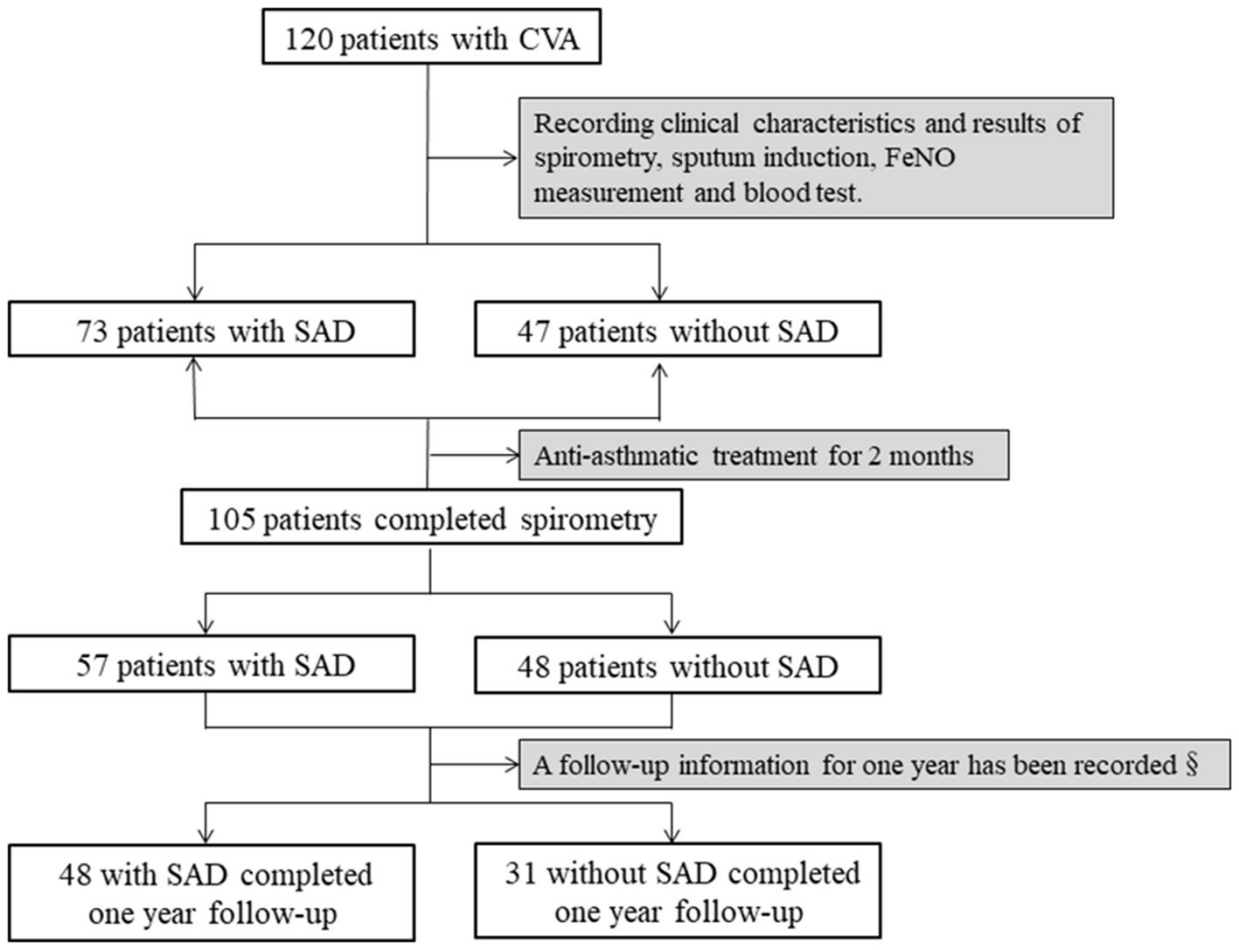
Study profile SAD was identified when any two parameters of MMEF% pred, FEF50% pred, and FEF75% pred were <65%. Antiasthmatic treatment included budesonide/formoterol (160/4.5 μg) alone, montelukast alone, or the combination of budesonide/formoterol and montelukast. SAD, small airway dysfunction; §, asthma medication of the patients was withdrawn after 2 months of treatment; MMEF, maximal mid-expiratory flow; FEF50%, forced expiratory flow at 50% of forced vital capacity; FEF75%, forced expiratory flow at 75% of forced vital capacity.

This was a retrospective observational study. The demographics and baseline clinical characteristics of all patients, and the results of their spirometry and bronchial provocation tests were recorded. Sputum induction for differential cells and FeNO were measured in 112 and 116 patients, respectively, at baseline. Asthma medicines, such as ICS/LABA alone, LTRAs alone, or the combination of ICS/LABA plus LTRAs, were prescribed to all patients. After treatment for 2 months, the cough visual analog scale (VAS) was evaluated in all of the patients, and spirometry was repeated in 105 patients. Meanwhile, sputum induction and FeNO measurements were performed repeatedly in 98 and 101 patients, respectively. A total of 79 patients with complete cough relief began treatment withdrawal after 2 months (48 patients with SAD and 31 patients without SAD), and they completed the follow-up for 1 year; the follow-up information regarding cough relapse and wheezing development was recorded. The study profile is shown in Figure 1. SAD was defined as any two parameters of maximal mid-expiratory flow (MMEF)% pred, forced expiratory flow at 50% of forced vital capacity (FEF50%) pred, and forced expiratory flow at 75% of forced vital capacity (FEF75%) pred measuring <65% (Xiao et al., 2020). The study was approved by the Ethics Committee of the First Affiliated Hospital of Guangzhou Medical University (Number: 202018).

### Assessment and Measurements

The VAS was used to assess cough severity, which was assessed on a scale of 0–10, and was based on the severity of the cough. A score of 0 meant “absolutely no cough,” whereas a score of 10 denoted “the most serious cough” (Lai et al., 2018). Sputum was induced and processed as described in our previous study (Yi et al., 2016). Briefly, sputum induction was performed by using nebulization of hypertonic saline; a cell smear was stained with hematoxylin-eosin, and a differential cell count was obtained by counting 400 non-squamous cells. The FeNO measurement was performed in accordance with the standard procedure described in American Thoracic Society (ATS) and European Respiratory Society (ERS) (American Thoracic Society European Respiratory Society, 2005). Briefly, subjects were instructed to inhale deeply via a mouthpiece and then exhaled with a constant flow (0.05 L/s) for 10 s. Spirometry and bronchial provocation tests were performed as recommended by the ATS (Miller et al., 2005). The provocative cumulative dose of methacholine causing a 20% fall in FEV_1_ (PD20-FEV_1_) ≤ 12.8 μmol was adopted as bronchial hyper-responsiveness.

### Statistical Analysis

Statistical analyses were conducted by using SPSS (version 18.0). Cough duration, VAS, percentages of sputum eosinophil, and FeNO were expressed as the median and interquartile range (IQR), and spirometry parameters were presented as the mean ± SD. A Mann-Whitney test was used to compare the cough VAS, sputum Eos%, and FeNO, and two independent sample *t*-tests were used to compare the FEV_1_% pred, FEV_1_/FVC, MMEF% pred, FEF50% pred, FEF75% pred, PEF% pred, and percentages of blood eosinophils (Eos%) among groups. The two related-samples *t*-tests and Wilcoxon test were used, respectively, to compare the FEV_1_% pred, FEV_1_/FVC, MMEF% pred, FEF50% pred, FEF75% pred, PEF% pred, cough VAS, sputum Eos%, and FeNO before and after the treatment. A *p* < 0.05 was considered statistically significant.

## Results

### Demographics and Baseline Features

A total of 120 patients with CVA were enrolled in our study. Among them, 85 (70.8%) patients were women, and the mean age of all patients was 39.4 ± 12.7 years. Of the 120 patients, 73 patients (60.8%) presented with SAD. Compared with patients without SAD, patients with SAD showed a significantly higher ratio of women (78.1 vs. 59.6%, *p* = 0.029), older mean age (41.9 vs. 35.4, *p* = 0.005), and longer median cough duration (24.0 vs. 6.0, *p* = 0.031), and a significantly lower FEV_1_% pred, FEV_1_/FVC, MMEF% pred, FEF50% pred, FEF75% pred, PEF% pred, and PD20 (all *p* < 0.01). However, no significant differences were found in cough VAS (*p* = 0.682), percentages of sputum eosinophil (*p* = 0.765), blood Eos% (*p* = 0.790), FeNO (*p* = 0.964), and total IgE (*p* = 0.131) between patients with SAD and patients without SAD (Tables 1, 2).

**Table 1:** Demographics and baseline clinical characterization.

	**Total**	**Patients with SAD**	**Patients without SAD**
*N* (%)	120	73 (60.8)	47 (39.2)
Female, %	70.8	78.1	59.6^*^
Age, years	39.4 ± 12.7	41.9 ± 12.0	35.4 ± 12.7**^#^**
Duration, mon	12.0 (4.0–48.0)	24.0 (4.0–60.0)	6.0 (3.0–24.5)^*^
AR, %	51.7	51.4	52.2
VAS	5.0 (5.0–7.0)	5.0 (5.0–7.3)	5.0 (5.0–7.0)
TIgE, KU/L (*n* = 101)	139.4 (48.6–258.5)	157.5 (55.9–517.5)	135.0 (31.6–252.0)
Blood Eos%	5.2 ± 4.0	5.2 ± 4.2	5.3 ± 3.7
Sputum Eos% (*n* = 112)	8.2 (3.1–28.0)	8.4 (2.8–32.8)	8.0 (3.3–27.3)
FeNO (*n* = 116)	58.0 (24.0–98.0)	60.0 (25.0–93.8)	51.0 (23.0–100.0)

*Data were presented as % or mean ± SD or median (IQR). SAD, small airway dysfunction; AR, allergic rhinitis; VAS, visual analog scale; TIgE, total immunoglobulin E; FeNO, fractional exhaled nitric oxide. Patients with SAD vs. patients without SAD*:

* *p < 0.05*;

# *p < 0.01*.

**Table 2:** Baseline spirometry parameters in patients with SAD and patients without SAD.

	**Total**	**Patients with SAD**	**Patients without SAD**
FEV_1_% pred	93.6 ± 11.97	88.7 ± 10.1	101.1 ± 10.8^**#**^
FEV_1_/FVC	78.4 ± 8.6	74.1 ± 6.4	85.0 ± 7.5^**#**^
MMEF% pred	61.7 ± 20.6	48.6 ± 10.6	82.1 ± 15.0^**#**^
FEF50% pred	66.7 ± 21.5	53.8 ± 12.9	86.8 ± 16.2^**#**^
FEF75% pred	56.2 ± 24.9	41.8 ± 12.9	78.3 ± 22.6^**#**^
PEF% pred	94.8 ± 13.7	90.2 ± 13.4	101.8 ± 11.0^**#**^
PD20, μmol	2.5 (0.5–6.9)	1.2 (0.4–3.8)	4.7 (1.5–10.0)^**#**^

*Data were presented as % or mean ± SD or median (IQR). SAD, small airway dysfunction; FEV1, forced expiratory volume in 1 s; MMEF, maximal mid-expiratory flow; FEF50%, forced expiratory flow at 50% of forced vital capacity; FEF75%, forced expiratory flow at 75% of forced vital capacity; PEF, peak expiratory flow. Patients with SAD vs. patients without SAD*:

# *p < 0.01*.

### SAD After Antiasthmatic Treatment and Follow-Up

**Figure 2:**
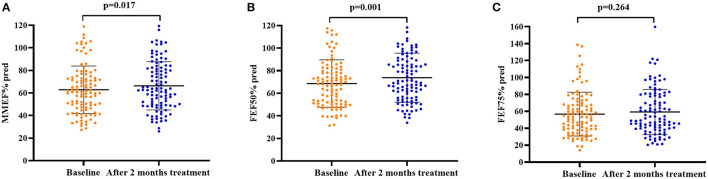
Spirometry parameters of small airway function at baseline and after 2 months of antiasthmatic treatment in 105 patients with CVA. **(A)** MMEF% pred at baseline and after 2 months of antiasthmatic treatment; **(B)** FEF50% pred at baseline and after 2 months of antiasthmatic treatment; **(C)** FEF75% pred at baseline and after 2 months of antiasthmatic treatment. Bars indicate the mean ± SD. MMEF, maximal mid-expiratory flow; FEF50%, forced expiratory flow at 50% of forced vital capacity; FEF75%, forced expiratory flow at 75% of forced vital capacity; SAD, small airway dysfunction.

After 2 months of antiasthmatic treatment, repeat spirometry was performed in 105 patients, among whom, 57 (54.3%) patients still presented with SAD. There was no significant change in the prevalence of SAD after 2 months of treatment as compared with the baseline (54.3 vs. 60.8%, *p* = 0.321). For all patients, the cough VAS (median from 5.0 to 1.0, *p* = 0.000), sputum eosinophils count (from 8.2 to 4.3%, *p* = 0.000), and FeNO level (from 58.0 to 36.0 ppb, *p* = 0.000) decreased significantly while the PEF% pred (mean value from 95.2 to 98.9%, *p* = 0.001), MMEF% pred (mean value from 62.9 to 66.4%, *p* = 0.017), and FEF50% (mean value from 68.6 to 73.8%, *p* = 0.001) increased significantly after 2 months of treatment (Figure 2).

**Figure 3:**
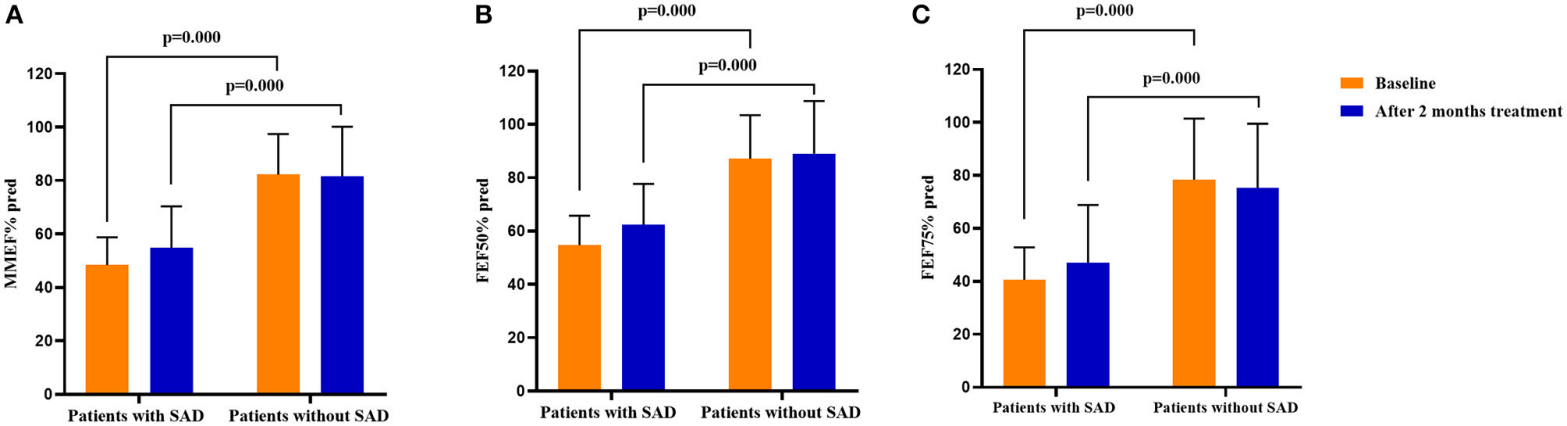
Comparison of the spirometry parameters on small airway function in patients with SAD and patients without SAD at baseline and after 2 months of treatment. **(A)** The MMEF% pred in patients with SAD and patients without SAD at baseline and after 2 months of treatment; **(B)** The FEF50% pred in patients with SAD and patients without SAD at baseline and after 2 months of treatment; **(C)** The FEF75% pred in patients with SAD and patients without SAD at baseline and after 2 months of treatment. SAD, small airway dysfunction. Bars indicate the mean ± SD. MMEF, maximal mid-expiratory flow; FEF50%, forced expiratory flow at 50% of forced vital capacity; FEF75%, forced expiratory flow at 75% of forced vital capacity; SAD, small airway dysfunction.

Additionally, significant decreases in cough VAS, sputum eosinophils counts, and FeNO levels were found in both patients with SAD and patients without SAD when the 2 months of treatment was finished (all *p* < 0.05). The FEV_1_% pred (*p* = 0.001), FEV_1_/FVC (*p* = 0.002), PEF% pred (*p* = 0.000), MMEF% pred (*p* = 0.000), FEF50% pred (*p* = 0.000), and FEF75% pred (*p* = 0.000) were improved significantly in patients with SAD, while no improvement in the parameters of small airway function was found in patients without SAD. After 2 months of treatment, there was no significant difference in cough VAS, sputum Eos%, and FeNO between patients with SAD and patients without SAD, but the levels of FEV_1_% pred, FEV_1_/FVC, PEF% pred, MMEF% pred, FEF50% pred, and FEF75% pred were still significantly lower in patients with SAD than in patients without SAD (all *p* < 0.01). The changes in cough VAS, sputum Eos%, and FeNO level from baseline to withdrawal of antiasthmatic treatment showed no significant differences between patients with SAD and patients without SAD (Table 3; Figures 2, 3).

**Table 3:** Clinical and pathophysiological parameters in patients with SAD and patients without SAD at baseline and after antiasthmatic treatment for 2 months.

**Variables**	**Patients with SAD**		**Patients without SAD**	
	**Baseline**	**After treatment**	**Baseline**	**After treatment**
VAS	5.0 (5.0–7.3)	1.0 (0.0–2.3)^**#**^	5.0 (5.0–7.0)	1.5 (0.0–3.0)^**#**^
Sputum Eos% (*n* = 98)	8.4 (2.8–32.8)	5.1 (1.0–12.2)^**#**^	8.0 (3.3–27.3)	3.4 (0.6–9.8)^**#**^
FeNO (*n* = 101)	60.0 (25.0–93.8)	36.0 (20.0–59.0)^**#**^	51.0 (23.0–100.0)	35.0 (19.0–52.8)^**#**^
FEV_1_% pred (*n* = 105)	88.7 ± 10.1	92.4 ± 11.1^**#**^	101.1 ± 10.8	99.3 ± 11.9
FEV_1_/FVC	74.1 ± 6.4	76.6 ± 6.9^**#**^	85.0 ± 7.5	84.4 ± 6.9
MMEF% pred	48.6 ± 10.6	54.9 ± 15.5^**#**^	82.1 ± 15.0	81.6 ± 18.5
FEF50% pred	53.8 ± 12.9	62.4 ± 15.3^#^	86.8 ± 16.2	89.0 ± 19.8
FEF75% pred	41.8 ± 12.9	47.1 ± 21.8^#^	78.3 ± 22.6	75.5 ± 24.2
PEF% pred	90.2 ± 13.4	95.7 ± 14.8^**#**^	101.8 ± 11.0	103.2 ± 13.8

*Data were presented as % or mean ± SD or median (IQR). SAD, small airway dysfunction; FeNO, fractional exhaled nitric oxide; FEV_1_, forced expiratory volume in 1 s; MMEF, maximal mid-expiratory flow; FEF50%, forced expiratory flow at 50% of forced vital capacity; FEF75%, forced expiratory flow at 75% of forced vital capacity; PEF, peak expiratory flow. Baseline vs. After 2 months of treatment*:

# *p < 0.01*.

The demographics and baseline clinical characteristics between patients followed up for a year and those who were not followed up were comparable (Table 4). Among the patients with a follow-up of a year, as compared with the patients without SAD, the patients with SAD showed no significant difference in the rate of relapse (50.0 vs. 41.9%, *p* = 0.483), and we noted a trend, albeit not statistically significant, for a higher rate of wheeze development (10.4 vs. 0%, *p* = 0.063) during follow-up. In addition, no significant difference was shown in the median onset time of cough relapse from the treatment withdrawal between the patients with SAD [1.8 m (IQR: 1.0–3.0)] and the patients without SAD [1.7 m (0.8–5.9)] (*p* = 0.586).

**Table 4:** Demographics and baseline clinical characterization between patients with follow-up and patients without follow-up.

	**Patients with follow-up**	**Patients without follow-up**	***P***
*N*	79	41	NS
Female, %	70.9	70.7	0.986
Age, years	38.8 ± 12.3	40.4 ± 13.5	0.213
Duration, mon	12.0 (3.0–36.5)	21.0 (5.0–64.5)	0.113
AR, %	46.2	62.5	0.093
VAS	5.0 (5.0–7.0)	5.0 (4.8–7.0)	0.250
TIgE, KU/L (*n* = 101)	131.5 (44.2–339.8)	214.9 (67.0–404.4)	0.167
Blood Eos%	5.5 ± 4.0	4.7 ± 4.1	0.640
Sputum Eos% (*n* = 112)	10.0 (3.0–28.4)	7.5 (3.3–20.4)	0.590
FeNO (*n* = 116)	52.5 (28.0–88.3)	74.0 (22.0–109.5)	0.454
FEV_1_% pred	93.7 ± 11.8	93.4 ± 14.0	0.557
FEV_1_/FVC	77.9 ± 7.8	79.3 ± 10.1	0.168
MMEF% pred	61.9 ± 20.8	61.2 ± 20.5	0.712
FEF50% pred	68.1 ± 21.1	65.5 ± 19.9	0.750
FEF75% pred	55.4 ± 25.1	57.9 ± 24.5	0.939
PEF% pred	95.3 ± 14.5	93.7 ± 12.2	0.280
PD20, μmol	1.8 (0.4–6.9)	3.0 (0.8–7.4)	0.485

*Data were presented as % or mean ± SD or median (IQR). SAD, small airway dysfunction; AR, allergic rhinitis; VAS, visual analog scale; TIgE, total immunoglobulin E; FeNO, fractional exhaled nitric oxide; FEV1, forced expiratory volume in 1 s; MMEF, maximal mid-expiratory flow; FEF50%, forced expiratory flow at 50% of forced vital capacity; FEF75%, forced expiratory flow at 75% of forced vital capacity; PEF, peak expiratory flow*.

## Discussion

This study found that more than half of the patients with CVA had SAD, showed a longer duration of disease and worse pulmonary ventilation function, but had similar cough severity and airway eosinophilic inflammation to those patients without SAD. SAD persisted despite improvement in lung function and airway inflammation after short-term antiasthmatic treatment for 2 months.

The definition and diagnosis criteria of SAD are still controversial and diverse. At present, gold standard parameters for the diagnosis of SAD are still warranted. Techniques available for assessment of SAD include spirometry, impulse oscillometry (IOS), imaging, etc. (Bickel et al., 2014; Mcnulty and Usmani, 2014). Among those, IOS is a non-invasive and reproducible method, by which R_5−20_ has been considered as an index for measuring SAD. However, IOS has not been wildly applied in clinical practice due to equipment and technical restraint. As another non-invasive measure of airway morphologies, imaging can separate different disease phenotypes. However, direct imaging measurement of small airways is difficult as they are largely beyond the resolution of CT scanners. The expensive cost and special software requirements have also limited its clinical applications. As a non-invasive and easy-to-perform measurement, the spirometry test has been widely applied in clinical practice. Among the spirometry parameters, FEF25–75% pred, FEF50% pred, and FEF75% pred have been used to reflect SAD, especially FEF25–75% (Marazzini et al., 1989; Pellegrino et al., 2005; Fujisawa et al., 2013; Pisi et al., 2013; Mcnulty and Usmani, 2014; Yuan et al., 2019). Furthermore, a recently published study with a large sample suggested that FEF25–75% was a more sensitive measure reflecting SAD in patients with asthma (Qin et al., 2021). The cut-off points of middle expiratory flows for defining SAD were not compatible in different studies (Marazzini et al., 1989; Usmani et al., 2016; Yuan et al., 2019), however, the value of FEF25–75% pred (MMEF% pred) <65% has been used widely in finding small airway disease in previous research studies or guideline recommendations (Marazzini et al., 1989; Pellegrino et al., 2005; Pefura-Yone et al., 2014; Yi et al., 2020; Qin et al., 2021). In our study, SAD was indicated when any two parameters of MMEF% pred, FEF50% pred, and FEF75% pred were <65%, which mainly according to the SAD definition were used by previous studies aiming at the Chinese adult population with CVA or asthma (Gao et al., 2020; Xiao et al., 2020) and the recommendation of the Guidelines for Lung Function Examination in China [Pulmonary Function Workgroup of Chinese Society of Respiratorydiseases (CSRD) Chinese Medical Association, 2014].

Small airway dysfunction is commonly present in asthmatic patients at a prevalence ranging from 40 to 70% in different studies (Tunon-De-Lara et al., 2007; Anderson et al., 2012; Usmani et al., 2016). The various prevalence of SAD may be dependent on the use of different evaluation methods, definition standards, and disease severity among different studies. The investigation conducted by Yuan et al. (2019) showed that the incidence of small airway disease determined by FEF50% pred <70% in patients with CVA was 45.4%, which was lower than that in our results. We identified better lung function and milder eosinophilic inflammation of the enrolled patients in Yuan's study, and the therapeutic condition of these patients was unclear before they were included in that study, which might have resulted in the difference from our study.

Asthmatic patients with SAD showed specific clinical relevance, and the correlations between small airway function and airway inflammation, disease severity, and asthma control have been reported largely across patients with classic asthma (Battaglia et al., 2005; Bourdin et al., 2006; Manoharan et al., 2014; Abdo et al., 2020). Studies have indicated that blood eosinophilia (Kjellberg et al., 2016), smoking history, and low FEV_1_ are risk factors for peripheral airway dysfunction in asthmatic patients. In addition, a correlation of small airway function and poor asthma control (Farah et al., 2012; Manoharan et al., 2014), sputum eosinophil counts, or FeNO has been found in asthmatic patients (Battaglia et al., 2005), and even a positive correlation between sputum neutrophils and SAD was found in stable asthma (Manoharan et al., 2014; Contoli et al., 2015; Farah et al., 2015; Cottini et al., 2020). However, our study included corticosteroid-naïve CVA patients without smoking history and showed similar cough VAS scores, percentages of sputum eosinophils, and blood eosinophil counts between patients with SAD and patients without SAD. It seemed that the presence of SAD would not yield a difference in cough severity and airway inflammation in patients with CVA.

However, patients with CVA who presented with SAD were older and had a trend of longer cough duration and worse large airway function regarding a lower level of FEV_1_% pred and FEV_1_/FVC. It has been reported that disease duration and age are non-physiological variables that are significantly correlated with SAD (Farah et al., 2015; Postma et al., 2019). Older age and longer cough duration might result in greater impairment of small airway function.

The treatment of CVA was similar to that of classic asthma (Lai et al., 2018). Similar to findings reported by other studies (Chung and Pavord, 2008; Liu et al., 2012; Tagaya et al., 2015), our results also showed that cough VAS was significantly improved in all patients after 2 months of standard antiasthmatic treatment, and the percentages of sputum eosinophils and FeNO levels were significantly decreased. Sugawara et al. (2019) found that the improvements in symptoms of patients with CVA were closely correlated with the ICS particle size, but it was unclear in terms of the prognosis. Better correlations between small airway function and ACT and a reduction in exacerbations have also been found in patients with eosinophilic asthma under anti-T2 biological therapy (Abdo et al., 2020). However, in our study, there were no significant differences in the change in cough VAS, sputum eosinophils, or FeNO levels between patients with SAD and those without SAD from baseline to 2 months after the completion of treatment, suggesting that small airway function might not play a role in the treatment response regarding the improvement in cough symptoms and airway inflammation in CVA patients with a regular ICS particle size. Although there was no significant improvement in lung function in asthmatic patients without SAD after antiasthmatic treatment, all large airway function indexes, such as FEV_1_% pred and FEV_1_/FVC, and small airway functions, such as MMEF% pred and FEF50 pred, were significantly improved in patients with SAD, which may be due to their lower lung function than that in patients without SAD. It has been reported that an extra-fine inhaler could lead to an improvement in SAD and clinical outcomes and better asthma control in asthmatic patients (Vos et al., 2013). In our study, the proportion of SAD showed no significant changes compared with the baseline, with 54.3% of patients with CVA still presenting with SAD after 2 months of treatment, and worse small airway function was still found in patients with SAD after treatment, indicating that conventional particle ICS/LABA or short-term antiasthmatic treatment could not reverse SAD. Whether long-term treatment or ultrafine particle inhalers will improve the small airway function and prognosis of patients with CVA needs further study.

A medical history of repeat admissions to hospitals or emergency departments, recent exacerbations, specific concomitants, frequent use of inhaled short-acting β2 agonists, use of systemic corticosteroids, higher levels of blood or sputum eosinophils, and increased FeNO are potential predictors of asthmatic exacerbation (Mccarren et al., 1998; Bousquet et al., 2005; Miller et al., 2006; Chipps et al., 2012; Papaioannou et al., 2016; Kimura et al., 2018). Studies reported in recent years have showed that small airway function is also correlated with the number of asthmatic exacerbations (In 'T Veen et al., 2000; Bourdin et al., 2006), and impairment of distal airways increases the risk of disease exacerbation. Higher airway hyper-responsiveness, eosinophilia, and atopy are risk factors for CVA to develop into classic asthma (Kim et al., 2003; Fujimura et al., 2004; Takemura et al., 2007). Whether patients with SAD are more likely to develop classic asthma is not clear. We found that patients with SAD had a longer duration and lower PD20 provoked by methacholine, which indicated greater hyper-responsiveness, suggesting that patients with CVA who had SAD might be more likely to develop classic asthma. Our preliminary data showed that when compared with the patients without SAD, the patients with SAD showed no significant difference in the rate of relapse, but we noted a trend, albeit not statistically significant, for a higher rate of wheeze development (10.4 vs. 0%, *p* = 0.063) during the follow-up. We speculated that significant differences might be shown after a further longer-term follow-up. However, the relevance of SAD and relapse or asthma development should be confirmed in future prospective studies with a reasonable design.

There were also some limitations in our study. Firstly, although FEF25–75% pred was the most commonly used index indicating small airway function, the definition of SAD in this study was based on pulmonary ventilation function indicators (MMEF% pred, FEF50% pred, and FEF75% pred), which is a functional definition. Failure to further confirm such findings in combination with IOS or imaging changes may cause these results to not reflect the real proportion of SAD in patients with CVA. However, based on previous studies and considering clinical practice, it is more direct, convenient, and simple to evaluate the small airway function of patients by the pulmonary function index, furthermore, in an observational study, Pisi et al. revealed that FEF25–75% was significantly correlated with R_5_-_20_ in asthmatic patients, indicating its potential utility in predicting SAD (Pisi et al., 2013), and our findings of SAD prevalence in patients with CVA were similar to a previous report aiming at patients with CVA (Gao et al., 2020), suggesting that these spirometry parameters can give an indication of SAD for our enrolled patients. Additionally, this was a retrospective study rather than a prospective investigation, and the relationship between SAD and the prognosis of patients with CVA was derived from speculation according to our preliminary data, which still needs further prospective research in the future.

## Conclusions

Over half of patients with CVA presented with SAD. Compared with patients without SAD, patients with SAD were older and showed worse large airway function, but had similar cough severity and airway inflammation. Although CVA patients with SAD showed improvements in cough VAS scores, sputum eosinophil counts, FeNO levels, and lung function, SAD still persisted after antiasthmatic treatment. SAD may play a significant role in the prognosis of CVA.

## Data Availability Statement

The raw data supporting the conclusions of this article will be made available by the authors, without undue reservation.

## Ethics Statement

The studies involving human participants were reviewed and approved by the Ethic Committee of the First Affiliated Hospital of Guangzhou Medical University (Number: 202018). The patients/participants provided their written informed consent to participate in this study.

## Author Contributions

KL and FY: conception and design of the study. FY, ZJ, HLi, CG, and HLu: data collection, data analysis, interpretation, and revising of the submitted manuscript. FY: drafting the manuscript. WL and QC: sputum induction and differential cell count. KL: a critical review of the manuscript. All authors contributed to the article and approved the submitted version.

## Funding

This study was funded by Chiesi and the Key-Area Research and Development Program of Guangdong Province (2019B020227006) and the National Natural Science Foundation of China (NSFC) (82000024). Chiesi, as a study sponsor, provided the funding source in the development of the study but did not play a role in the study design, data analysis, data interpretation, or writing of the report.

## Conflict of Interest

The authors declare that the research was conducted in the absence of any commercial or financial relationships that could be construed as a potential conflict of interest.

## Publisher's Note

All claims expressed in this article are solely those of the authors and do not necessarily represent those of their affiliated organizations, or those of the publisher, the editors and the reviewers. Any product that may be evaluated in this article, or claim that may be made by its manufacturer, is not guaranteed or endorsed by the publisher.

## Abbreviations

CVA: cough variant asthma
SAD: small airway dysfunction
VAS: cough visual analog scale
FeNO: fractional exhaled nitric oxide
ICS: inhaled corticosteroids
LABA: long-acting β2-agonist
LTRAs: Cysteinyl-leukotrienes receptor antagonists
FEV_1_: forced expiratory volume in 1 s
MMEF: maximal mid-expiratory flow
FEF50%: forced expiratory flow at 50% of forced vital capacity
FEF75%: forced expiratory flow at 75% of forced vital capacity
PEF: peak expiratory flow.
